# Recent Progress on MOF‐Derived Heteroatom‐Doped Carbon‐Based Electrocatalysts for Oxygen Reduction Reaction

**DOI:** 10.1002/advs.201700515

**Published:** 2017-12-05

**Authors:** Qian Ren, Hui Wang, Xue‐Feng Lu, Ye‐Xiang Tong, Gao‐Ren Li

**Affiliations:** ^1^ MOE Laboratory of Bioinorganic and Synthetic Chemistry The Key Lab of Low‐Carbon Chemistry & Energy Conservation of Guangdong Province School of Chemistry Sun Yat‐sen University Guangzhou 510275 China

**Keywords:** electrocatalysts, heteroatom‐doped carbon, metal‐organic frameworks, oxygen reduction reaction

## Abstract

The oxygen reduction reaction (ORR) is the core reaction of numerous sustainable energy‐conversion technologies such as fuel cells and metal–air batteries. It is crucial to develop a cost‐effective, highly active, and durable electrocatalysts for ORR to overcome the sluggish kinetics of four electrons pathway. In recent years, the carbon‐based electrocatalysts derived from metal–organic frameworks (MOFs) have attracted tremendous attention and have been shown to exhibit superior catalytic activity and excellent intrinsic properties such as large surface area, large pore volume, uniform pore distribution, and tunable chemical structure. Here in this review, the development of MOF‐derived heteroatom‐doped carbon‐based electrocatalysts, including non‐metal (such as N, S, B, and P) and metal (such as Fe and Co) doped carbon materials, is summarized. It furthermore, it is demonstrated that the enhancement of ORR performance is associated with favorably well‐designed porous structure, large surface area, and high‐tensity active sites. Finally, the future perspectives of carbon‐based electrocatalysts for ORR are provided with an emphasis on the development of a clear mechanism of MOF‐derived non‐metal‐doped electrocatalysts and certain metal‐doped electrocatalysts.

## 1 Introduction

In order to meet the urgent requirements for sustainable and renewable power supplies in the booming electronics industry, numerous endeavors have been made in the development of high‐efficiency energy conversion and storage devices.^1^, ^2^, ^3^, ^4^, ^5^, ^6^, ^7^, ^8^, ^9^, ^10^, ^11^ The oxygen reduction reaction (ORR) plays a key role in the progress of advanced electrochemical energy conversion systems, including proton exchange membrane fuel cells (PEMFCs), metal–air batteries, direct alcohol fuel cells (DAFCs), etc. Nevertheless, these energy systems have their own limitations, including the sluggish kinetics of ORR, high cost and poor stability because of the aggregation/dissolution of Pt nanoparticles (NPs) in the Pt‐based electrocatalysts of these energy conversion systems.^5^, ^12^, ^13^, ^14^, ^15^, ^16^ Developing highly efficient electrocatalysts for ORR is a crucial step to accelerate the commercialization of these advanced energy storage and conversion devices. In this connection, a series of relevant explorations have been performed in both electrocatalysts and support materials for ORR, including that: (i) alloying Pt with other transition metals to increase specific activity and reduce the cost of electrocatalysts;^17^, ^18^, ^19^, ^20^, ^21^, ^22^ (ii) developing highly active nonprecious metal or metal oxide electrocatalysts with low cost;^23^, ^24^, ^25^, ^26^ (iii) searching new‐type support materials to increase active site centers.^27^, ^28^, ^29^, ^30^, ^31^, ^32^, ^33^ Recently, the number of researches related to nonprecious metal electrocatalysts has greatly increased because of popular price, regulable morphological structure and high electrocatalytic activity compared with noble metal electrocatalysts, such as transition metal oxides,^34^, ^35^, ^36^, ^37^, ^38^, ^39^, ^40^ metal phosphides,^41^, ^42^, ^43^, ^44^ metal sulfides,^45^, ^46^, ^47^, ^48^ as well as carbon‐based materials.^49^, ^50^, ^51^, ^52^ Among these advanced electrocatalysts, porous carbons have been widely applied in many research fields as electrode materials and catalytic supports for energy storage devices, fuel cells, adsorbents, and drug delivery carriers, etc. They feature plentiful attractive properties including high surface area, high conductivity, low cost, abundant porosity, and excellent corrosion resistance, which can be considered as the most ideal supports or Pt‐free electrocatalysts for ORR in fuel cells.^53^, ^54^, ^55^, ^56^ There are various methods to obtain highly porous carbon‐based electrocatalysts, such as direct carbonization of polymeric aerogels, electrospinning fiber technique, pyrolysis of organic precursors with physical or chemical activation, and nanocasting with sacrificial solid templates (such as zeolites and mesoporous silicas).^57^, ^58^, ^59^ Although the activated porous carbons possess a high surface area, the disordered structures caused by broad pore size distribution may limit their availability. In consequence, it is essential to explore a proper precursor for the preparation of metal‐free carbon‐based electrocatalysts with high specific surface areas, large pore volumes, and proper chemical stabilities for ORR. MOFs as the novel precursors of highly porous carbon‐based electrocatalysts have attracted great concerns.^60^, ^61^, ^62^, ^63^, ^64^, ^65^, ^66^ Their unique intrinsic structures provide an opportunity to obtain higher surface areas (≈10 000 m^2^ g^−1^) than other porous materials, such as zeolites and activated carbon, etc.^67^ Distinguished from the harsh operating conditions and high energy consumption of traditional synthetic strategy, the carbon‐based materials from carbonized MOFs as electrocatalysts offer numerous advantages: (i) The structure, composition, and function of MOFs possess flexible tunabilities because they can be modularly designed according to the targeted properties by self‐assembly of metal ions/clusters and bridging organic ligands^68^, ^69^; (ii) It is easy to implement heteroatom‐doped carbon materials with different nonmetals or metal elements, and this can be attributed to the ultrahigh surface area, various pore size distribution, and ordered porous structure of MOFs, which are easy to adsorb organic molecules.^70^, ^71^, ^72^ In 2008, Xu and co‐workers demonstrated the first example that they employed MOF‐5 framework as a template to obtain nanoporous carbon, which displayed high electrochemical performance as the electrode material for electrochemical double‐layered capacitor (EDLC) due to the high surface area and hydrogen adsorption capacity of nanoporous carbon derived from MOF‐5.^70^

To improve electron transport property and ORR catalytic activity, single/dual heteroatom‐doped carbon‐based electrocatalysts have been widely fabricated, and they are segmented into two kinds of forms, namely, nonmetal‐doped porous carbon materials (e.g., N, S, B, and P) and metal‐doped porous carbon materials (e.g., Fe, Ni, Co, and Cu), and they exhibit more active sites and enhanced electrocatalytic activity for ORR.^73^, ^74^, ^75^, ^76^ Both theoretical and practical studies have verified that the intrinsic characteristics of functionalized carbon materials, such as inner microstructures and compositions, electronic features, surface and partial electrochemical properties, could be effectively adjusted by an appropriate incorporation of heteroatoms, which always results in a lower energy barrier of oxygen adsorption and activation or significantly improved electrocatalytic activity of ORR.^62^, ^73^, ^74^, ^75^ In terms of the structure of carbon‐based electrocatalysts, designing hierarchical nanopore structures can not only enhance the mass transport and expose more active sites for catalysis but also retain more electrolyte ions and high electrical conductivity. Furthermore, another effective strategy for highly active ORR electrocatalysts is to confine catalytic sites within a permeable but robust shell (such as highly porous carbon materials) and induce a localized high instantaneous concentration for the fast heterogeneous catalysis. Fortunately, MOF‐derived heteroatom‐doped carbon‐based electrocatalysts could be well designed to realize such a structure.^58^, ^59^, ^60^, ^61^ During a thermal activation process, organic linkers of MOF precursors will be converted into carbon, while the inherent frameworks will be maintained to form a highly porous structure of electrocatalysts, without any secondary carbon supports or pore forming agents. The as‐prepared MOF‐derived heteroatom‐doped carbon electrocatalysts always possess a high surface area, uniformly distributed active sites and an excellent electrical conductivity, which are beneficial to fast mass transport and electron transfer. In recent years, heteroatom‐doped MOF‐derived carbon‐based electrocatalysts has been developed rapidly. In this review, we mainly focus on the recent developments of heteroatom‐doped MOF‐derived carbon‐based electrocatalysts for ORR, including discussions on active site centers of nonmetal heteroatom‐doped carbon electrocatalysts, metal heteroatom‐doped carbon electrocatalysts, and the structure optimization.

## 2 MOF‐Derived Nonmetal Heteroatom‐Doped Porous Carbon Electrocatalysts

In recent years, nonmetal‐doped carbon‐based electrocatalysts have demonstrated an enormous research prospect for the development of ORR, especially nitrogen (N)‐doped carbon‐based catalysts. Researchers observed that the carbon catalysts containing nitrogen species, involving carbon nanotubes (CNTs),^77^, ^78^ graphene,^79^, ^80^ and hollow carbon spheres,^81^ exhibited good electrical conductivity and high selectivity toward ORR as an excellent nonmetal electrocatalyst candidate for ORR.^82^, ^83^, ^84^ This can be ascribed to the intrinsic electronic properties originating from the conjugation between graphene π‐system and nitrogen lone pair electrons.^85^ In 2009, Dai and co‐workers first engineered an aligned nitrogen‐containing carbon nanotube arrays as a metal‐free electrocatalyst for four‐electron oxygen reduction process in alkaline fuel cell, which showed a good catalytic activity, low overpotential and good electrochemical tolerance.^86^

### 2.1 N‐Doped Carbon Electrocatalysts

**Figure 1:**
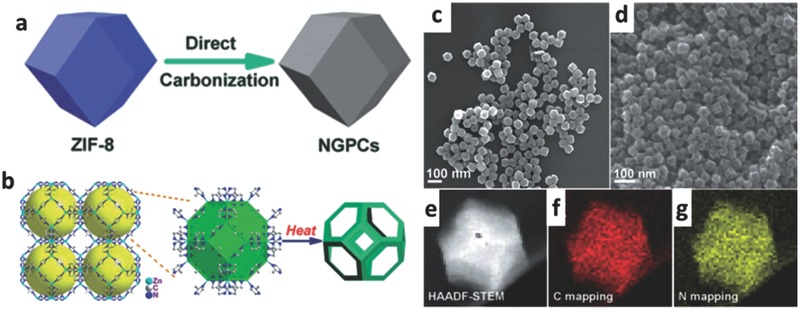
a,b) Schematic illustration of ZIF‐8‐driven template synthesis of highly graphitized nitrogen‐doped porous carbon nanopolyhedra; SEM images of c) monodispersed ZIF‐8 nanopolyhedra and d) NGPC‐1000‐10; e–g) HAADF–STEM images of a single carbon polyhedron and the corresponding C‐, and N‐elemental mappings. Reproduced with permission.^87^ Copyright 2014, Royal Society of Chemistry.

The methods for the synthesis of MOF‐derived N‐doped carbon electrocatalysts depend on the properties, existential form and type of doped element and the prospective properties of targeted electrocatalyst. The essential characteristics of MOF precursors, such as size dimension, topology, superficial features, pore volume, and aperture‐structure, are greatly related to the microscopic structure, morphology, spatial distribution of holes, and catalytic activity of the final carbon composite electrocatalysts derived from MOFs.^50^, ^77^, ^84^ Universal synthesis strategies for previous reports can be divided into two types: (i) direct carbonizing of nitrogen‐bearing MOFs precursor (e.g., ZIF‐8), which is called as “in situ” nitrogen doping; (ii) postsynthetic modification strategy of carbonification process, which can introduce nitrogen into catalysts by using a nitrogen‐containing guest molecule (e.g., NH_3_, acetonitrile, dicyandiamid). MOFs could be used as an immediate precursor to construct heteroatom‐doped porous carbon electrocatalysts without any additional secondary carbon source or a pore‐forming agent, which can be attributed to their well‐defined porous structure, and the rich carbon content of organic building blocks. If the organic ligands of MOFs contain various heteroatoms, the doping of heterogeneous atoms is easy and cost‐effective to be implemented, which could be used as ideal precursors to produce carbon‐based electrocatalysts with uniformly distributed catalytic centers and high density active sites. In the direct carbonizing growth, MOFs as the carbon and nitrogen sources are usually used as a self‐sacrificing template, while the doping of carbon with nitrogen takes place during the conversion of carbon nanostructures from a nitrogen‐bearing MOF precursor. For example, Hong and co‐workers^87^ presented a highly porous zeolite‐type MOF (ZIF‐8) as both the precursor and template, which turned to be chemically and thermally stable electrocatalysts. The aromatic methyl‐imidazole ligand of ZIF‐8 is oxygen‐free and the abundant nitrogen is directly incorporated into aromatic ring, which could promote the consolidation of highly enriched nitrogen‐containing active sites into carbon matrices. In their research, nitrogen‐doped carbon nanopolyhedra were obtained with a highly graphitic carbon skeleton via a high‐temperature (1000 °C) process, during which Zn ions in ZIF‐8 were reduced to metal Zn and vaporized (boiling point 908 °C), and the possible residuals were removed by a following acid treatment (**Figure** **1**a,b). The resultant N‐doped graphitic porous carbons present a uniform rhombic dodecahedra with a relatively uniform N distribution (shown in Figure 1c–g) and possess a hierarchical porous structure, high active site density, and uniform distribution of active sites. The optimized electrocatalyst (NGPC‐1000‐10) displayed a superior electrocatalytic activity and cycling stability for four‐electron‐dominant ORR process and splendid methanol tolerance in alkaline media, which can be ascribed to the collaborative role of high surface area and porosity, abundant active sites stemming from high graphitic‐N portion, and high degree of graphitization.

However, most of carbon‐based ORR electrocatalysts derived from MOF are generally subjected to contamination of metal species (e.g., Fe, Zn, or Co), which leads to not only a metal corroding in acidic media, but also an ambiguous catalytic mechanism of heteroatom‐doped carbon electrocatalysts for ORR. For example, David Lou et al. reported a N‐doped carbon nanotube frameworks (NCNTFs) with hierarchical shells of interconnected crystal derived from the direct carbonization of ZIF‐67.^88^ The NCNTFs demonstrated a superior electrocatalytic activity and durability as an ORR electrocatalyst compared with a commercial Pt/C electrocatalyst. However, the existent of Co nanoparticles encapsulated by multilayer carbon shells might lead to an adverse mechanism of pure metal‐free carbon catalyst due to metal leaching or etching in alkaline or acidic electrolytes after long‐time electrochemical processes. In view of the above reasons, several additional steps have been performed to remove the residual metal or metallic impurities in carbon electrocatalysts, such as treating them with a highly corrosive acid (e.g., HF) and aqueous alkali.^68^, ^72^ Although these methods have achieved an obvious effect for the production in nonmetal carbon electrocatalyst, the HF would bring about physical discomfort to human beings, or cause environmental contaminations. Hence, it is necessary to explore a universally applicable and environment‐friendly preparation method for high‐performance nonmetal porous carbon electrocatalysts toward ORR. Fortunately, Cao and co‐workers found that N‐doped porous carbon electrocatalysts could be obtained by introducing additional carbon source‐glucose into the carbonation process of host ZIF‐7 framework [Zn(benzimidazole)_2_(H_2_O)_3_], which was green and environment friendly. Glucose not only played a critical role in the complete removal of residual Zn metal or zinc compounds but also enhanced the graphitization degree of ultimate carbon materials due to the addition of secondary carbon sources.^89^

**Figure 2:**
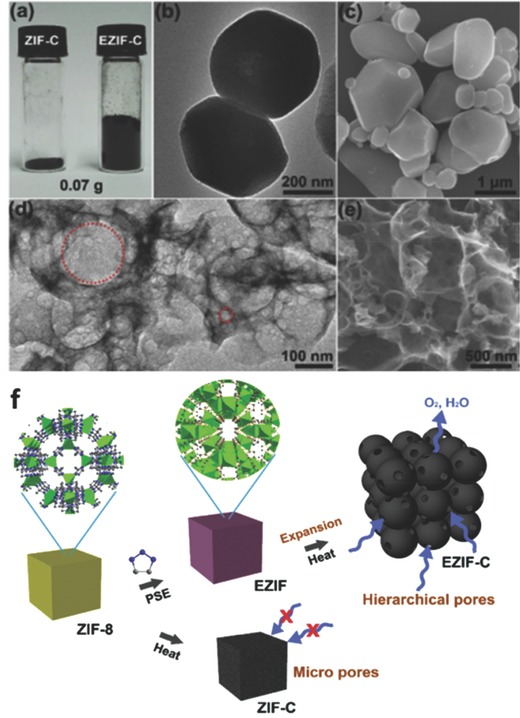
a) Photograph of ZIF‐C and EZIF‐C with the same weight. b) TEM and c) SEM images of ZIF‐C; d) TEM and e) SEM images of EZIF‐C; f) The large and small red circles in (d) indicated the macropore and mesopore, respectively. The illustration for the formation of the hierarchically porous carbon from ZIF‐8. Reproduced with permission.^93^ Copyright 2017, Elsevier.

In addition to a direct carbonization of nitrogen‐bearing MOFs precursor, a postsynthetic modification strategy has also been adopted to prepare nitrogen‐containing porous carbon catalysts by using a MOF with nitrogen‐containing guest molecules (e.g., NH_3_, dicyandiamide, urea, acetonitrile) as nitrogen sources. Generally, N‐doped porous carbon materials can be successfully prepared via an arc‐discharge/vaporization approach, chemical vapor deposition (CVD), or plasma treatment under the presence of nitrogen species atmosphere such as NH_3_, pyridine, or acetonitrile.^90^, ^91^, ^92^ Nevertheless, they usually need to sustain rigorous reaction conditions and complex multiple step procedures, even leading to high energy consumption. In addition, they might suffer from poor chemical homogeneity and reproducibility. For MOFs used as hard templates for preparing porous carbon, a new member of polyporous solid matrices should possess an abundantly well‐defined pore structure, good chemical tunability, and a high specific surface area, which could admit a number of nitrogen‐containing guest molecules into the holes of host MOF framework to obtain uniform doped and nitrogen‐containing porous carbon catalysts. Excitingly, some nitrogen‐rich guest molecules can not only provide additional nitrogen source for MOF‐derived porous carbon electrocatalysts, but also play a role of “explosive” in the carbonization process of host MOFs framework. Inspired by this idea, in a research by Zou and co‐workers,^93^ 1,2,3‐triazole was introduced into the microporous MOF (ZIF‐8) precursor (denoted as EZIF) as the pore‐expansion agent to functionalize and prepare the N‐doped foam‐like carbon material, via a solvent‐assistant‐linker‐exchange method (as shown in **Figure** **2**). Triazole derivatives also have been confirmed to be newfangled high‐nitrogen energetic compounds, including triazole of a five‐membered heterocyclic compound with three nitrogen atoms. In the thermal activation process of a precursor, the eruption of a huge amount of miscellaneous gas (such as CO_2_, NO_2_, NO, HCN, and N_2_) can lead to a remarkably enlarged surface area (1257 m^2^ g^−1^) and high pore volume (4.4 cm^3^ g^−1^) with coexistences of micropore, mesopore, and macropores because of the decomposition of “explosive” (1,2,3‐triazole).^94^, ^95^, ^96^ On account of these unique structural merits, the optimized catalyst (EZIF‐C) shows a superior catalytic performance towards ORR in both acidic and alkaline electrolytes.

**Figure 3:**
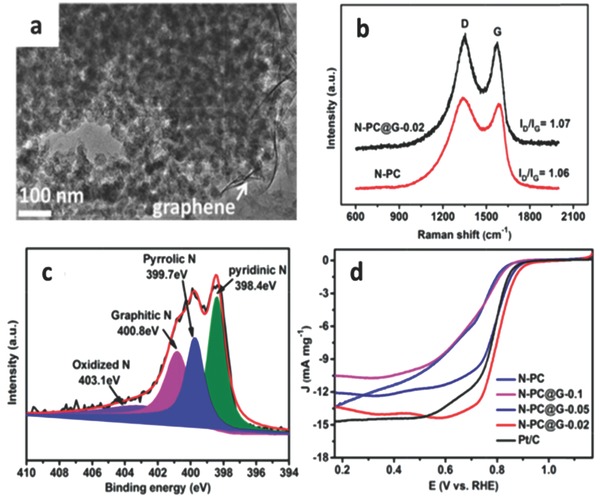
a) TEM image of as‐prepared N‐PC@G‐0.02 composite. b) Raman spectra of the prepared N‐PC@G‐0.02 and N‐PC samples. c) High‐resolution N1s XPS spectra of N‐PC@G‐0.02 sample. d) LSV curves of ZIF‐8 derived carbon and commercial Pt/C electrocatalysts in O_2_‐saturated 0.1 m KOH solution at a scan rate of 10 mV s^−1^ and a rotating rate of 1600 rpm. Reproduced with permission.^100^ Copyright 2016, Elsevier.

For N‐doped carbon electrocatalyst materials, the content and form of C and N play a significant role in efficient ORR catalysis. Generally speaking, different forms of nitrogen and carbon will produce synergistic effects for advanced catalysis process.^104^ Relevant researches show that the highly graphitized carbon could achieve superior catalytic activity toward ORR.^92^, ^97^, ^98^, ^99^ Additionally, ambiguous or multifarious types of carbons can collectively achieve a synergistic effect for ORR catalysis. For instance, Cai and co‐workers have successfully synthesized a sandwich‐like N‐doped porous carbon@graphene composite (N‐PC@G) that derived from a ZIF‐8@graphene oxide composite (**Figure** **3**), whose high catalytic activity could be attributed to the synergistic effect between ZIF‐8 derived carbon porous carbon and graphene.^100^ Among the three types of N species (pyridinic‐, pyrrolic‐, and quaternary‐ N), the planar structured pyridinic and pyrrolic forms are more favorable for highly active ORR catalytic process compared with the 3D structured quaternary N species.^101^, ^102^, ^103^ To some extent, exploring applicable strategies to increase the content of pyridinic‐ and pyrrolic‐nitrogen of N‐doped carbon electrocatalysts is in turn beneficial to improve the catalytic activity for ORR.^104^, ^105^

### 2.2 Binary or Ternary Nonmetallic Heteroatom‐Doped Carbon‐Based Electrocatalysts

**Figure 4:**
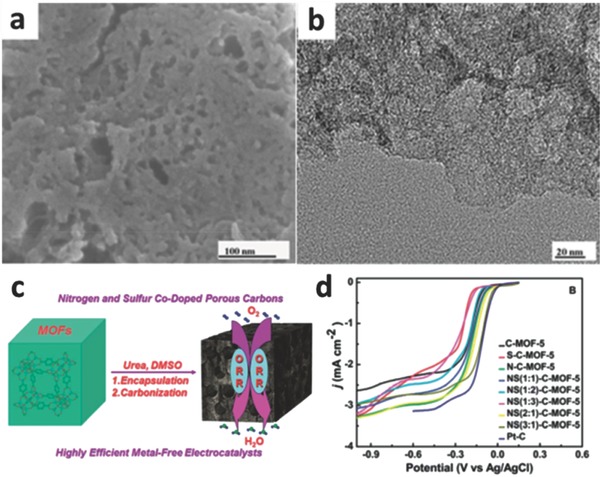
a) SEM and b) HRTEM images of the typical NS (3:1)‐C‐MOF‐5 electrocatalyst; c) synthesis procedure for MOF‐5 templated N and S co‐doped porous carbon as metal‐free electrocatalysts for ORR; d) LSVs of different samples (different atomic ratio of N and S) at 1600 rpm. Reproduced with permission.^114^ Copyright 2014, Royal Society of Chemistry.

Because of larger asymmetrical spin and the optimized charge density in carbon skeletons, heteroatom‐doped carbon‐based catalysts will generate a potential synergistic effect among various heteroatoms. The multiplex heteroatom‐doped carbon‐based catalysts can offer further improved electrocatalytic activity for ORR.^106^, ^107^, ^108^ Considerable researches have testified the efficient ORR catalytic activities of heteroatom (such as N, S, B, P)‐doped carbon electrocatalysts stemming from its distinct electronegativity, which can notably modify their electronic properties and chemical characteristics.^109^, ^110^, ^111^, ^112^ Among the heteroatoms, S (electronegativity: 2.58) atom possesses the outstanding synergetic effect and potential to replace C atom when it co‐dops with N.^107^ This is mainly ascribed to the combination of sulfur‐related active sites and hierarchical porous textures, which felicitates the fast diffusion of oxygen molecules and electrolyte ions to catalytic sites and the release of products from the sites. In comparison with the traditional CVD technique using liquid organics, S‐containing functionalities can be incorporated into sp^2^ hybrid of carbon atoms by a direct pyrolysis procedure of host MOFs with S‐containing guest molecules, and the latter can gain a superior homogeneity and higher S content. For instance, Gao et al. reported the formation of S‐doped grapheme/Cu using liquid S‐hexane mixture vapor at 950 °C, causing the existence of S atoms in the linear nanodomains with a low content (0.6%) other than as doping S atoms.^113^ Dai and co‐workers developed MOF‐5 precursors by encapsulating urea and dimethyl sulfoxide (DMSO) (as N and S precursors, respectively) into the hole of host MOF via a carbonization at 900 °C in ultrapure N_2_ (**Figure** **4**). The characterization results demonstrated that N and S atoms had been incorporated into the skeletons of porous carbon, and the optimized electrocatalyst (NS(3:1)‐C‐MOF‐5) was composed of 88.85% C, 3.31% N, and 1.08% S. The ultimate nitrogen and sulfur co‐doped porous carbon electrocatalyst (NS(3:1)‐C‐MOF‐5) showed a more positive onset potential (−0.005 V vs. Ag/AgCl), long‐term stability, and excellent methanol crossover resistance in 0.1 m KOH due to the synergistic effect of the co‐doped N and S, which was even comparable to commercial 20% Pt/C electrocatalyst.^114^

**Figure 5:**
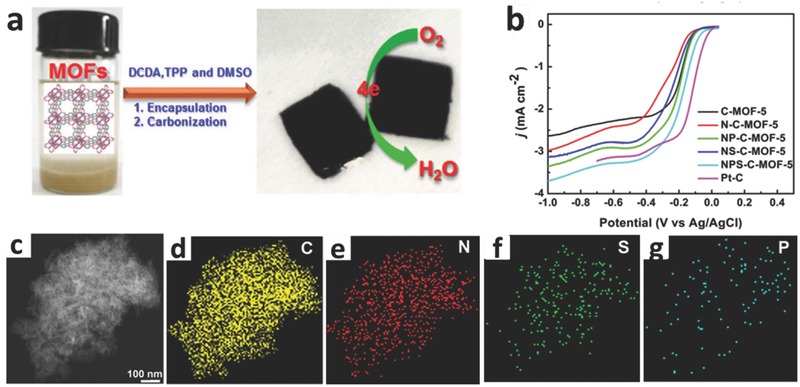
a) Schematic illustration of the synthesis of MOF‐templated NPS‐C‐MOF‐5 as a metal‐free electrocatalyst for the ORR; b) LSVs of different samples at a rotation rate of 1600 rpm; c) STEM images of NPS‐C‐MOF‐5; d–g) the corresponding C‐, O‐, N‐, P‐, and S‐elemental mappings, respectively. Reproduced with permission.^115^ Copyright 2014, the authors, published by Nature Publishing Group.

Moreover, the nitrogen (N), phosphorus (P), and sulfur (S) ternary co‐doped porous carbon electrocatalysts (named as NPS‐C‐MOF‐5) towards ORR have been further fabricated using MOF‐5 as a self‐sacrificing template. Dicyandiamid (DCDA), triarylphosphine (TPP), and dimethyl sulfoxide (DMSO) were introduced as N, P, and S precursors, and the uniform dopings of N, P, and S into the resultant carbon materials were realized (**Figure** **5**). When the heteroatoms (N, P, S) are doped into carbon skeletons, the pore structure of these materials is changed, and it can be conjectured that part of micropores of heteroatom‐doped materials can be sintered, further reducing the ratio of micropores and increasing the ratio of mecropores, which may causes different ORR activities. On account of the synergistic effects of N, P, and S ternary‐doped, the NPS‐C‐MOF‐5 electrocatalyst shows a more positive onset potential (about −0.006 V at 1600 rpm), and demonstrates a superior electrocatalytic activity, excellent methanol tolerance and outstanding long‐term stability for ORR in 0.1 m KOH compared with other carbon electrocatalysts.^115^

**Figure 6:**
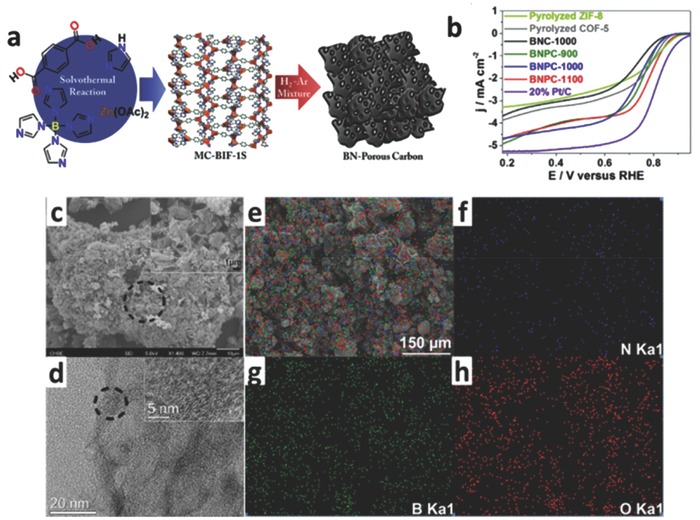
a) The synthetic scheme of BNPCs; b) LSVs of BNC, BNPCs, and non‐B‐doped carbon at 1600 rpm; c) SEM images and d) TEM images of BNPC‐1000; e–h) EDS mapping of BNPC‐1100 and the corresponding, N, B, and O elemental, respectively. Reproduced with permission.^122^ Copyright 2017, Elsevier.

In recent years, boron‐doped (B‐doped) carbon electrocatalysts have been also applied to ORR. Boron (electronegativity: 2.04) is one electron deficient and is less electronegative than carbon. Boron would be a good candidate, and it is similar to nitrogen when it induces an electronegativity difference in carbon materials. The electronic structure of the carbon support will be changed when electron‐rich N and electron‐deficient B are simultaneously doped into carbon lattice, and then will affect the ORR activity considerably.^116^, ^117^, ^118^ A recent first‐principles molecular dynamics (MD) study has explored the ORR activity of B–N–C motifs located at the edges of graphene. In acidic media, the edge motifs of B, C, N were demonstrated to promote the reduction of O_2_ to H_2_O.^119^ In a research finished by Feng and co‐workers, the density functional theory (DFT) calculations demonstrated that the increased electrocatalytic active sites for ORR could be attributed to the chemisorbing of O_2_ on graphitic BN_3_ sites. Furthermore, DFT calculations have demonstrated that B sites could improve the adsorption of intermediates for ORR in the B, N co‐doped graphene electrocatalyst, such as *OOH.^119^, ^120^, ^121^ Therefore, B, N co‐doped graphitizing carbon electrocatalysts not only possess excellent electrocatalytic activity for ORR, but also demonstrate an extremely high selectivity (nearly 100%) for the four‐electron ORR pathways.^120^, ^121^ Compared with the conventional doping approaches, MOFs as a precursor to prepare N, B co‐doped carbon materials could precisely control the distribution of N, B species and ensure that the doped sites can be distributed evenly in carbon skeletons. For example, Zhao et al. prepared a bifunctional B, N co‐doped highly porous carbon (BNPC) electrocatalyst for ORR and OER via a direct pyrolysis method by using the MOFs containing Zn, N, and B (MC‐BIF‐1S) as precursor under the atmosphere of H_2_/Ar mixture. Zn was reduced and then was evaporated during the process of pyrolysis, and N and B were kept in the carbon skeleton, resulting in a N, B dual‐doped metal‐free carbon electrocatalyst. EDS elemental mapping characterization of the optimized BNPC‐1100 showed the uniform distributions of the doped B and N elements in carbon frameworks (as shown in **Figure** **6**). As an important parameter, the effect of pyrolysis temperature to ORR catalytic activity was explored. Among diverse catalysts obtained by the different pyrolysis temperatures, the BNPC‐1100 (pyrolysis temperature: 1100 °C) showed the highest onset potential and the best kinetics (*E*_onset_ = 0.894 V and *E*_half wave_ = 0.803 V, vs. RHE). The outstanding oxygen catalytic activity of BNPC electrocatalysts can be attributed to the unique electronic properties resulting from synergistic effects of N, B, C components, as well as the large surface area, higher porosity and high content of pyridinic N.^122^

## 3 MOF‐Derived Metal Heteroatom‐Doped Porous Carbon Electrocatalysts

Among the numerous heteroatom‐doped carbon electrocatalysts, the transition metal (such as Fe, Co, Cu, Ni, Mn, etc.) and N co‐doped carbon catalysts can achieve high ORR catalytic activity owing to their unique electronic structures and synergetic effects between diverse active species. However, besides Co or Fe‐doped carbon electrocatalysts, MOF‐derived other metal, such as Cu, Ni, and Mn, doped carbon electrocatalysts are rarely published in recent years,^7^, ^123^, ^124^, ^125^ likely due to their poor catalytic activity and stability compared with Fe and Co‐doped catalysts.^126^, ^127^, ^128^, ^129^, ^130^, ^131^, ^132^, ^133^, ^134^, ^135^, ^136^ Hence, MOF‐derived Fe and Co‐doped carbon catalysts are mainly summarized in this review. The ORR activity of Co phthalocyanine complexes were firstly discovered by Jasinski in 1964,^137^ and then the concept was proposed by Yeager and co‐workers who handled the surfaces of carbon materials with nitrogen groups to bind transition metal species to form M–N*_x_* structure.^102^ Because of the similar M–N*_x_* catalytic sites, the M–N–C ORR electrocatalysts, which are usually obtained via a pyrolysis process of composite precursors containing metal ion, nitrogen, and carbon, have been extensively investigated.^138^, ^139^, ^140^ Different from most of the complex classical synthetic procedures, MOFs, as the solid precursors to form the M–N*_x_* moieties doped carbon electrocatalysts, possess three main advantages: (i) it is capable to form hierarchical pores by the pyrolysis of MOFs^141^, ^142^, ^143^, ^144^, ^145^; (ii) the high homodispersity of active metal centers in carbon matrices is beneficial for enhancing graphitization, as well as further promoting the electrocatalytic activity^146^, ^147^, ^148^, ^149^; (iii) the electrocatalytic properties can be regulated and controlled through a rational design of MOF precursor's composition and structure, or introducing guest molecules.^150^, ^171^, ^172^, ^186^, ^190^ In addition, in virtue of the protection of graphitic carbon shells around the metallic nanoparticles, the active metal centers in the carbon matrices of MN*_x_*/C structure are relatively stable. In view of three phase interfaces (gas, liquid, and solid) in the electrocatalytic process for ORR, it is imperative to prepare electrocatalysts with a hierarchically porous structure and uniformly distributed high‐density active sites to compensate low catalytic activity and avoid overcommitting the electrocatalysts, thus ensuring a high ORR performance.^151^, ^152^

### 3.1 Co–N*_x_* Doped Porous Carbon Electrocatalysts

**Figure 7:**
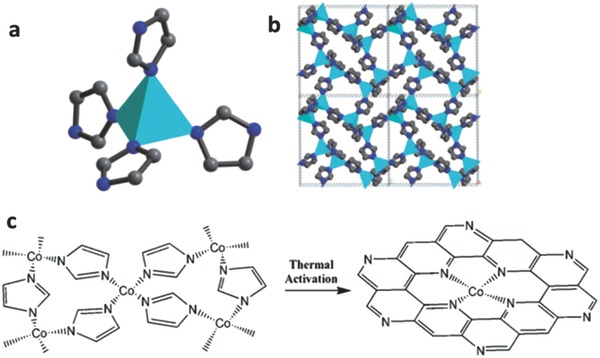
a) Local Co–N_4_ coordination moiety and b) structure packing of cobalt imidazolate framework along [100] direction (color scheme: turquoise = Co, blue = N, gray = C); c) the proposed structural conversion from cobalt imidazolate framework to the catalytic active site. Reproduced with permission.^154^

In 2006, Bashyam and Zelenay had employed polypyrrole as a matrix to capture Co atoms, wherein the Co atoms are linked to pyrrole units, generating active sites (Co–N) for ORR. Introducing polypyrrole into the framework of ORR electrocatalysts was parallel to the atomic configuration in cobalt porphyrins for the formation of CoN*_x_* active site. Cobalt–polypyrrole composites were synthesized on Vulcan XC 72 carbon (Co–PPY–C) via a simple chemical method, which allowed the direct formation of Co–N sites without resorting to pyrolysis. As a new nonprecious composite electrocatalyst, the Co–PPY–C composite with a high Co content shows a good catalytic activity and excellent stability for ORR in acidic medium.^153^ Hence, CoN*_x_* moieties as the active center for ORR have been widely studied. The zeolite imidazole framework is usually selected as the host MOF framework for purpose of CoN*_x_* moieties. Liu and co‐workers reported a CoN_4_/C electrocatalyst for ORR via pyrolysis of MOF framework, which was synthesized by 3,5‐imidazolate and Co(NO_3_)_2_·6H_2_O under solvothermal conditions (DMF). In CoN_4_/C electrocatalyst, each Co atom is coordinated with four nitrogen atoms from the neighboring imidazolate ligands, and CoN_4_ moieties are evenly distributed in the frameworks (shown in **Figure** **7**). The imidazolate groups will be converted into carbonaceous forms in the thermal activation process, which will remain as organic moiety (500 °C), carbonaceous (750 °C), and graphitic (900 °C) forms with the activation temperature increasing, while a portion of the nitrogen moieties will be reserved and will be coordinated with Co species in favor of forming catalytic sites for ORR. This research demonstrated the first Co–N_4_ moieties electrocatalyst derived from cobalt imidazole frameworks through thermal activation towards ORR.^154^

**Figure 8:**
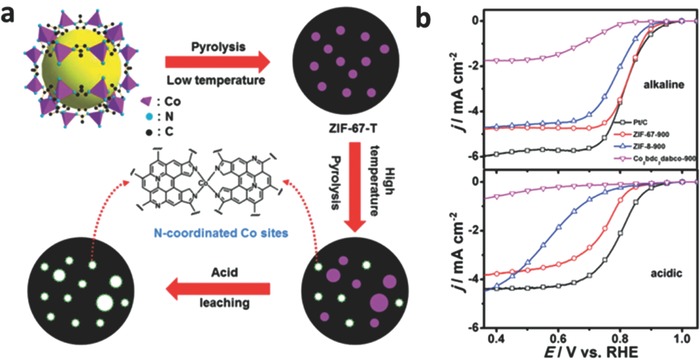
a) Schematic illustration of synthesis process for ZIF‐67‐T electrocatalysts. The coordination environment of Co in the product (a proposed structure) is also shown (a); b) Linear scan polarization curves of the ZIF‐67‐T samples and the acid leached sample ZIF‐67‐900‐AL in O_2_‐saturated 0.1 m KOH and 0.5 m H_2_SO_4_ (0.1 m HClO_4_ for Pt/C), measured on a RDE at 1600 rpm with a scan rate of 10 mV s^−1^. Reproduced with permission.^155^ Copyright 2014, Royal Society of Chemistry.

Subsequently, the ORR activity parameters are found that not only be bound up with the metal/ligand incorporation in MOF skeletons but also rely on the density of active sites of electrocatalysts. Hence, it is essential to form high density active sites and assemble proper M, N, and C components for high ORR performance. In previous researches, the active sites of electrocatalysts for ORR derived from three different kinds of MOFs (ZIF‐67, ZIF‐8, Co_2_(bdc)_2_(dabco); bdc = 1,4‐benzenedicarboxylate; dabco = 1,4‐diazabicyclo[2.2.2]‐octane) have been explored.^155^ The electrocatalyst derived from ZIF‐67 exhibits the best performance in both acidic (0.5 m H_2_SO_4_) and alkaline (0.1 m KOH) electrolytes among the three electrocatalysts. The coordination of Co metal and aromatic nitrogen (2‐methylimidazole) ligand in ZIF‐67 framework mimics the active CoN*_x_* moieties, and Co species contributes to the formation of active sites in the pyrolysis process. It is suggested that the well‐defined metal centers in MOF structures could be utilized to obtain ORR electrocatalysts with excellent performance. And the density of exposed CoN*_x_* sites is increased by the acid leaching process. The optimized electrocatalyst (derived from ZIF‐67) exhibits more positive half‐wave and onset potentials, and higher saturation current density compared with commercial Pt/C electrocatalysts in alkaline electrolytes, as well as better tolerance to methanol crossover and stability in both alkaline and acidic electrolytes (as shown in **Figure** **8**).^155^

**Figure 9:**
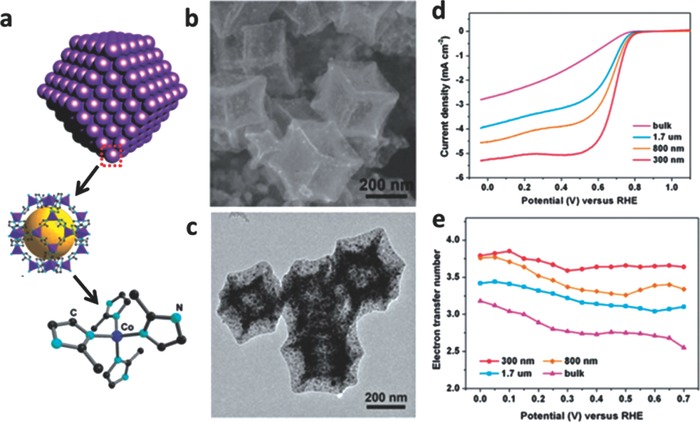
a) Structure information of ZIF‐67 crystal; b) SEM and c) TEM images of the sample after heat treatment at 750 °C; d) RDE polarization curves of 300 nm, 800 nm, 1.7 mm and bulk MDCs in 0.1 m HClO_4_ at a rotation rate of 1600 rpm. e) The electron transfer number as a function of potential for MDCs with various sizes. Reproduced with permission.^156^ Copyright 2014, Royal Society of Chemistry.

In addition to the influence of metal/ligand incorporation, the size dimension of electrocatalysts is also closely associated with the transport properties and electrocatalytic activity for ORR. Recently, Zou et al. reported that the size‐controlled monodispersed ZIF‐67‐derived electrocatalysts were obtained by simple adjustment of the reaction temperature and solvents without additional modulators (e.g., polymers, surfactants).^156^ With reaction temperature rising, the particle size of ZIF‐67 increases due to a further aggregation and growth of the initially formed crystal nucleus at higher temperature as shown in **Figure** **9**. As the size increases, the electrocatalysts showed the decreased catalytic activity for ORR. The electrocatalyst with the smallest particle size exhibits the highest ORR onset potential (*E*_onset_ = 0.86 V), the nearly highest electron transfer number (average value 3.7) and superior stability in acidic medium. The research implies that the electrocatalyst with smaller size could provide a more favorable mass and electron transfer process due to the easily accessible catalytic active site centers. Likewise, the influence of activation temperature in the process of electrocatalyst preparation is also explored, which is a key factor for the catalytic activity of ORR. With activation temperature increasing, the content of graphitic carbon and graphitic N climbs, and this is beneficial for the catalytic activity of ORR. However, in the above process, the overall nitrogen content in the sample declines because of the instability of nitrogen at elevated temperatures. The nitrogen species and content are very important for the formation of Co–N_4_ moieties in controlling the catalytic activity. Thus, a proper heat activation temperature is crucial for preparing high‐performance electrocatalysts for ORR.

**Figure 10:**
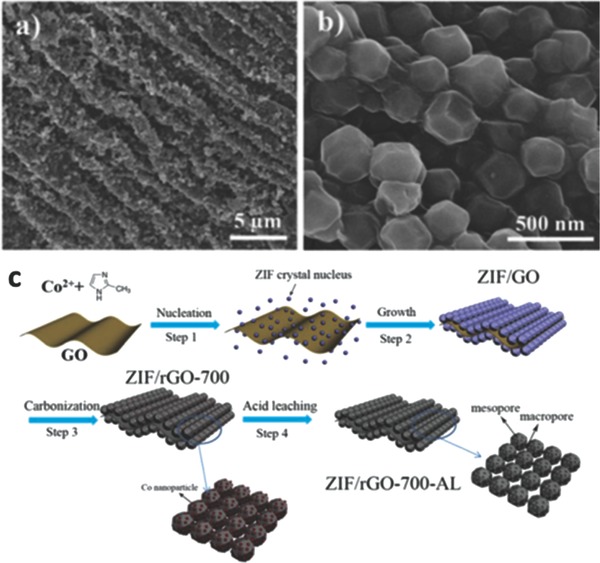
a,b) SEM images of ZIF/GO with different magnifications; c) The synthesis procedure of hierarchically nanoporous Co–N*_x_*/C materials (ZIF/rGO‐700‐AL). Step 1, the nucleation of ZIF seeds on both sides of a graphene oxide (GO) nanosheet. Step 2, the growth of ZIF nanocrystal arrays supported on a GO nanosheet (ZIF/GO). Step 3, carbonization of GO supported ZIF nanocrystal arrays at 700 °C in an Ar atmosphere to obtain a macroporous Co–N*_x_*/C material (ZIF/rGO‐700). Step 4, acid leaching to remove large Co nanoparticles and hence produce a meso/microporous structure (ZIF/rGO‐700‐AL). Reproduced with permission.^171^ Copyright 2015, Royal Society of Chemistry.

During the formation of Co–N*_x_* moieties, the structure‐directing agents are usually introduced to control the unique structure and porosity of Co–N_x_/C electrocatalysts for superior ORR catalytic activity. Certain special structures (such as hollow structure,^157^, ^158^, ^159^, ^160^, ^161^, ^162^, ^163^ yolk–shell^164^, ^165^, ^166^, ^167^, ^168^, ^169^, ^170^) of electrocatalysts can offer numerous benefits for catalytic performance. ZIF‐67 is usually selected as the host MOFs for the derived Co–N*_x_*/C electrocatalyst because of its abundant Co–N_4_ coordinate moieties and higher degree of graphitization than zinc‐based ZIF‐8 after a high temperature carbonization. For instance, graphene oxide (GO) can be used as a structure‐directing agent to fabricate ZIF‐67 nanocrystal arrays,^171^ and in the carbonation process it acts as a binder to connect the single derived carbon nanocatalyst into the macroporous structure and electrical conductor to increase the overall conductivity. Introducing hierarchical pore structure into Co–N*_x_*/C electrocatalysts by macroporous GO and meso/microporous ZIF‐derived carbon materials can not only enhance the porosity to expose more accessible active sites (Co–N_4_), but also bring a favorable electron transport performance (**Figure** **10**). This unique structure of Co–N*_x_*/C electrocatalyst is conducive to realize a superior ORR catalytic activity and stability in both alkaline and acidic media.

**Figure 11:**
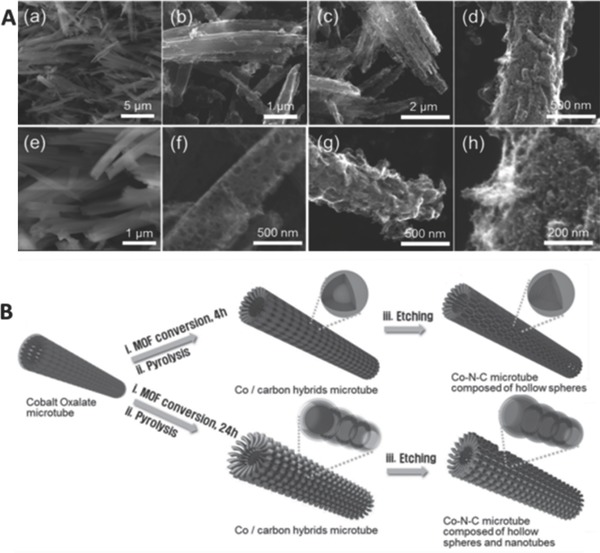
A) SEM images of a,e) cobalt oxalate microtubes and Co–N–C HMMTs with a controlled conversion reaction time of b,f) 4 h, c,g) 8 h, and d,h) 24 h; B) Schematic illustration of the synthesis method for the Co–N–C microtubular structure composed of hollow nanospheres and nanotubes. Cobalt oxalate microtube, which is used as both the solid precursor and the self‐template (a). Co/C hybrids microtube after a conversion reaction to ZIF‐67 microtube and a subsequent pyrolysis process at 800 °C for 3 h under argon flow (inset: inactive Co core@Co–N–C shells) (b). Co–N–C microtube composed of hollow subunits, nanopsheres, and nanotubes after an acid etching process (inset: Co–N–C hollow subunits) (c). Reproduced with permission.^172^

Analogously, cobalt oxalate microtubes (MTs) was used as a structure‐directing agent and solid cobalt precursor to controllably form a microtubular structure of Co–N*_x_*/C electrocatalyst, which is consisted of nanoscale nanotubes and spheres via an in situ conversion reaction and heat activation process (800 °C, 3 h).^172^ The conversion time of the cobalt oxalate to ZIF‐67 was tuned to control the morphology of Co–N*_x_*/C and growth rate of precursor (ZIF‐67) on the walls of cobalt oxalate MTs. With the conversion time increasing, the surface roughness and the amount of embedded Co–N*_x_* moieties in the graphite carbon matrices were significantly increased, resulting in the additional growth of tiny CNTs to form a hierarchical microtubular structure (as shown in **Figure** **11**). After removing the inactive Co and CoO*_x_* nanoparticles by an etching process, the hierarchical porous Co–N*_x_*/C electrocatalyst consisted of hollow nanospheres and nanotubes was obtained. At the same time, the recession of catalytic activity of Co–N*_x_*/C could be suppressed due to the unique hierarchical tubular structure, high degree of graphitization, numerous stable active sites, high porosity, and nanoscale open pores. The Co–N*_x_*/C electrocatalyst demonstrated a superior ORR activity with a high onset potential half wave potential, as well as excellent stability and tolerance for methanol crossover.

**Figure 12:**
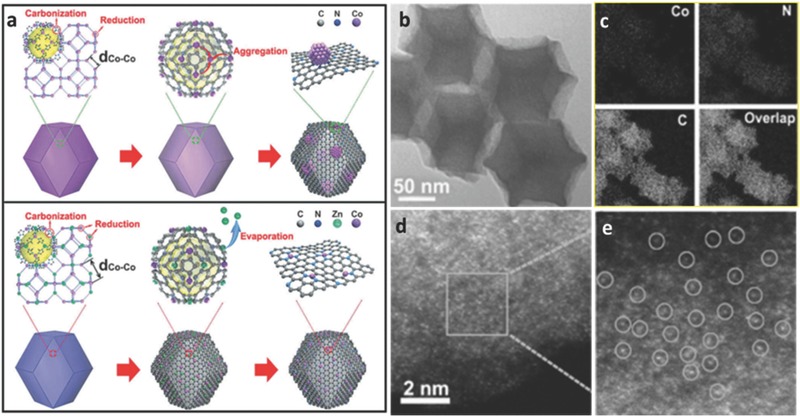
a) The formation of Co NPs–N/C (top) and Co SAs/N–C (bottom); b) TEM images of Co SAs/N–C(800); c) Examination of the corresponding EELS mapping reveals the homogeneous distribution of Co and N on the carbon support; d,e) Magnified HAADF–STEM images of Co SAs/N–C(800), showing that only Co single atoms are present in Co SAs/N–C. Reproduced with permission.^176^

Recently, single‐atom (SA) metal electrocatalysts have become a research hotspot in electrocatalysis due to their unexpected properties originating from atomic scale effects.^173^ But traditional researches on single‐atom electrocatalysts generally face certain difficulties: (i) to avoid the aggregation of metal atoms as well as assure a well‐defined single‐metal atoms on the incorporated support, an extremely low concentration of metal atoms is usually adopted, which might lead to a low density of metal active site centers;^174^, ^175^, ^176^, ^177^ (ii) in most instances, single‐atom metals may be confronted with markedly spatial inhomogeneity and poorly defined coordination environment.^173^, ^175^, ^176^ The metal in MOF nodes may be selectively replaced by other metals, and the coordination environment supplied by MOF linkers allows the space distance of reactive metal sites to be further tuned. The doped metals can be selectively removed, while the reactive metals can be reduced in situ. And then a metal SA electrocatalyst distributed in the porous carbon matrix can be obtained. For example, Li and co‐workers adopt a bimetallic ZIF (BIZIF) as the host MOF precursor, which contains evenly dispersed Zn^2+^, Co^2+^, and public ligands (2‐methylimidazole).^176^ In the construction of MOFs, Zn^2+^ was added to substitute a portion of Co^2+^ sites as a “fence” to further expand the spatial distance of adjacent Co atoms. The metal species (Zn and Co) were further reduced in situ to metallic cobalt and zinc nanoparticles in the presence of carbon. And then Zn atoms were evaporated away at 800 °C because of their low boiling point. Hence, a stable single atom Co/N‐doped porous carbon electrocatalyst with a high metal loading over 4 wt% was successfully obtained as shown in **Figure** **12**. The Co/N‐doped porous carbon electrocatalyst (Co–N*_x_*/C) exhibited a superior ORR catalytic activity (*E*_half wave_ = 0.881 V), outstanding thermal stability and chemical stability compared with commercial Pt/C. Furthermore, it was discovered that the Co–N*_x_* moieties and uniform N doped electrocatalysts derived from the BIZIF owned higher catalytic performance of ORR than Co–N*_x_*/C electrocatalysts derived from ZIF‐67 and metal‐free porous carbon electrocatalysts derived from ZIF‐8.

### 3.2 Fe–N Co‐Doped Porous Carbon Electrocatalysts

Hitherto, Fe–N co‐doped porous carbon electrocatalysts (Fe–N–C electrocatalysts) have been broadly regarded as one of the most advanced electrocatalysts for ORR in both alkaline and acid electrolytes, which can be ascribed to the coaction between iron species and N species in carbonaceous matrices.^178^, ^179^, ^180^, ^181^, ^182^ In the preparation of Fe–N–C electrocatalysts, iron salts, carbonaceous, and nitrogenous compounds are usually employed as precursors. For example, the Fe–N–C electrocatalysts were synthesized through hydrothermal method (180 °C for 15 h) with multiwalled CNTs as a carbon source, melamine as a nitrogen source and iron (III) nitrate nonahydrate providing iron species.^179^ A novel mulberry‐like Fe/N decorated carbon fibers were assembled by hollow mesoporous carbon spheres via a facile electrospinning method.^183^ The polyaniline, iron salt and urea were adopted as the precursors to prepare Fe–N–C electrocatalysts for ORR through a pyrolysis process.^184^ However, the homogeneity of Fe–N–C electrocatalysts are always poor because they may contain dual/multiple species of Fe‐based particles (e.g. Fe–N*_x_*, Fe_3_C@C, or metal Fe), which may hamper the in‐depth identification of the exclusive catalytic mechanism of each specie. Although Fe–N–C electrocatalysts show an outstanding catalytic activity for ORR under both acidic and alkaline conditions, the real catalytic active sites of Fe–N–C electrocatalysts are still debatable. In the report by Jiang and co‐workers,^179^ a Fe–N–C electrocatalyst was obtained through a pyrolysis of compounds consisted of multiwalled CNTs, melamine and iron (III) nitrate nonahydrate in argon flow. During the pyrolysis process, it was found that there was not only the production of Fe–N*_x_* coordination sites, but also the formation of graphene‐encapsulated metallic Fe/Fe_3_C nanocrystals. The in‐depth investigations on structure, composition, morphology, and electrochemical properties, unambiguously disclosed that the interaction between metallic iron and Fe–N_4_ coordination structure favoring the adsorption of oxygen molecule. Additionally, in order to avoid the interference of other sites, Joo et al. explored the roles of Fe–N*_x_* coordination and Fe–Fe_3_C@C sites in Fe–N–C electrocatalysts respectively through a novel method.^185^ The obtained electrocatalysts selectively contained Fe–N*_x_*, Fe–Fe_3_C@C, and N‐doped carbon (C‐N*_x_*) sites, respectively. The physicochemical characterizations and electrochemical investigations of three electrocatalysts demonstrated that Fe–N*_x_* sites catalyzed the four‐electron oxygen reduction for ORR, which acted a key role in the high ORR catalytic activity, while the Fe–Fe_3_C@C sites dominantly catalyzed the ORR via two electron (2 *e* ^−^) pathway and following 2 *e* ^−^ peroxide reduction, which played an auxiliary role in the ORR. Generally speaking, notwithstanding various active sites have been nominated for Fe–N–C electrocatalysts for ORR, the Fe–N*_x_* sites can be regarded as the predominant active centers, which can accelerate the dominant process of ORR catalytic activity (four electrons pathway).

**Figure 13:**
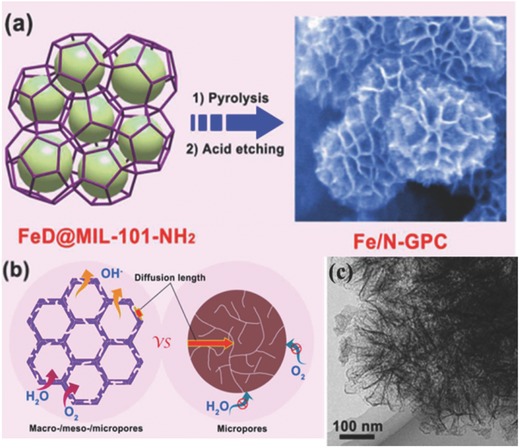
a) Schematic representation for the preparation of Fe/N‐GPC catalyst and b) the advantages of hierarchically porous architecture for promoting ORR; c) TEM image of Fe/N‐GPC. Reproduced with permission.^186^ Copyright 2017, American Chemical Society.

For the explorations of Fe–N–C electrocatalysts, it is still a challenge to design specific morphological structures and monodispersed active sites of Fe species in view of high reactivity by a high‐temperature pyrolysis. To further enhance ORR catalytic activity of Fe–N–C electrocatalysts, hierarchical pores and fully exposed active sites with high dispersity are concurrently designed to be incorporated into carbon skeletons. These features are expected to achieve the optimization of both functionalization surface and porous structure. Zou and co‐workers synthesized an atomically dispersed Fe/N‐doped hierarchical porous graphitic carbon via a double solvent method, which possesses a hierarchical pore structure and evenly dispersed Fe–N*_x_* active sites. MIL‐101‐NH_2_ was employed as the host MOF precursor due to its high surface area and large pore size, while FeCl_3_ and dicyandiamide with a high nitrogen content are selected to accommodate the catalytic function as Fe and N source as shown in **Figure** **13**. The optimized hierarchically graphitic porous carbon possessed high contents of Fe and N (with 1.1 wt% Fe and 3.3 wt% N incorporation), and demonstrated a comparable ORR catalytic activity with the commercial Pt/C and advanced non‐noble‐metal electrocatalysts under alkaline conditions. Benefiting from the unique atomically dispersed active sites and open structures, the electrocatalysts can expose to an electrochemical interface with high‐density accessible active sites, and generate an enhanced synergetic effect.^186^

**Figure 14:**
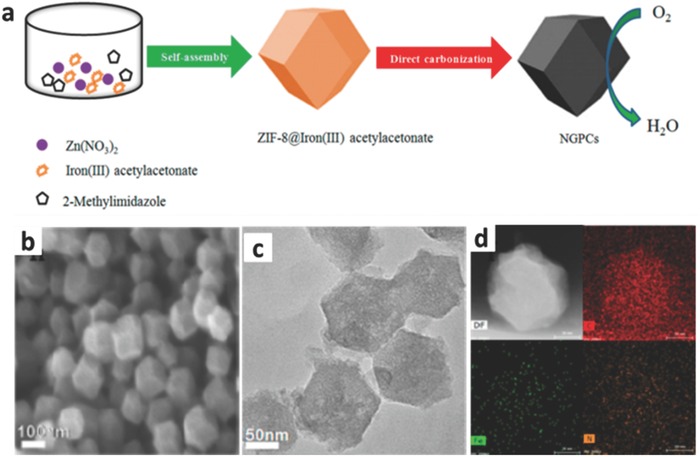
a) Schematic diagram showing the synthesis of Fe‐doped ZIF‐8 and the preparation of its derived doped carbon nanoframes; b) SEM image, c) TEM image, d) AADF–STEM, and elemental mapping images of C‐FeZIF‐900‐0.84. Reproduced with permission.^188^ Copyright 2017, American Chemical Society.

ZIF‐8 nanocrystals, as above mentioned, are the state of the art host MOF precursors to construct the heteroatom‐doped porous carbon electrocatalysts with a hierarchical porous structure and high degree of graphitization, because of their inherent high surface area and large pore volume. In view of this, a novel strategy for the construction of highly active Fe–N–C electrocatalysts with uniform Fe distribution at an atomic level has been demonstrated by Wu and co‐workers.^187^ Compared with traditional impregnation methods, Fe species were successfully incorporated into ZIF‐8 framework uniformly by partially replacing Zn ions during the synthesis process of precursor. Then the Fe–N–C electrocatalyst could be prepared by an one‐step thermal treatment of Fe/ZIF‐8 without additional posttreatment, in which the evaporation of zinc species leads to highly porous carbon matrices for the Fe–N–C electrocatalyst. Because of the obstruction of Zn ions, Fe species (Fe–N*_x_*) remained to be atomically dispersed without agglomeration into particles. Under acidic conditions, the Fe–N–C electrocatalyst exhibited a remarkable ORR catalytic activity with a high half‐wave potential and sufficient potential cycling stability. Different from other related researches, the Fe–N*_x_* doped carbon electrocatalysts kept a highly active catalysis for ORR without any graphitized carbons existing in carbon matrices, which may bring a new debate for the type of carbon in the high‐activity ORR electrocatalysis. In addition to effective structure design, the particle size of electrocatalyst is also worth to explore. Analogous to previous methods, the iron (III) acetylacetonate was introduced into the self‐assembly process of Zn^2+^ ions with 2‐methylimidazole to prepare Fe‐doped ZIF‐8, following a pyrolysis at 900 °C in an inert gas atmosphere.^188^ The investigation results showed that the initial well‐defined 3D polyhedron morphology of ZIF‐8 particles was preserved after incorporating Fe species with the adjustment of particle size. As the ratio of additive iron (III) acetylacetonate increased, the Fe‐doped ZIF‐8 crystals grew gradually. Such a phenomenon might be caused by the partial deprotonation of 2‐methylimidazole linkers with the presence of iron (III) acetylacetonate as shown in **Figure** **14**. Moreover, Fe doping was beneficial to achieve a good distribution of catalyst particle with an increased content of graphitic‐N and pyridinic‐N and a decreased content of oxidized‐N. In a word, the outstanding ORR activity and durability of Fe–N co‐doped carbon electrocatalysts can be ascribed to their high surface area, large porosity, uniform dispersion of Fe in nanoframes, and the enhanced proportion of active N species.

**Figure 15:**
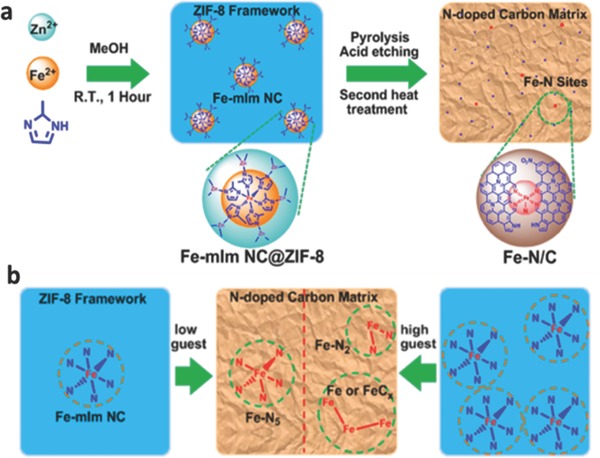
a) Illustration of the host–guest chemistry strategy to fabricate MOF‐derived Fe–N/C electrocatalysts; b) illustration of the host–guest roles on the states of Fe in the resulting Fe–N/C electrocatalysts. Reproduced with permission.^189^ Copyright 2017, American Chemical Society.

Furthermore, it was found that the electronic structure of Fe centers could be regulated by diverse configurations of Fe–N*_x_* MOFs. It has been proved that the different coordination states affect the adsorption and reduction of O_2_ and the binding energy of intermediates (OOH*, OH*), leading to the variations in catalytic activity of Fe–N/C electrocatalysts for ORR. As shown in Liang and co‐workers research,^189^ Fe–mIm nanocluster (NC) (guest)@zeolite imidazole (ZIF‐8) (host) precursors were designed and prepared through in situ introducing Fe^2+^ into the methanolic solution containing Zn^2+^ and 2‐mIm during the construction of ZIF‐8 framework. During the subsequent thermal decomposition at 900 °C, a significant host–guest‐dependent confinement effect was discovered between ZIF‐8 host network and guest Fe–mIm NC, and the adjustment of molar ratio of Fe^2+^/(Fe^2+^+Zn^2+^) resulted in different coordination states (two‐ to five‐coordinated configurations) of Fe–N*_x_* sites as shown in **Figure** **15**. The ORR electrocatalytic performances of three typical Fe–N*_x_* models with different coordination numbers (N–Fe–N_4_, Fe–N_4_, and Fe–N_2_) were deeply discussed. The results showed that the N–Fe–N_4_ (five‐coordinated) owned excellent electrocatalytic activity and selectivity for ORR in acidic medium, which promoted oxygen reduction rate via narrowing the reaction energy barrier and reducing the adsorption energy of intermediate OH.

**Figure 16:**
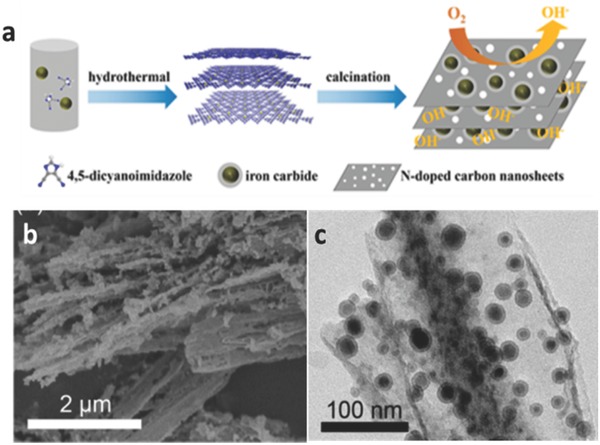
a) Schematic Illustration of the preparation of Fe_3_C@NC/NCS Nanohybrids; b) SEM image; c) TEM image of DCI‐Fe‐700 electrocatalyst. Reproduced with permission.^190^ Copyright 2017, American Chemical Society.

For the advanced ORR electrocatalysts, they usually possess high‐density active sites with a uniform distribution and hierarchical pore structure. Designing special porous structures of Fe–N/C electrocatalysts has proved to be an effective strategy. In some way, it is one of the most effective approaches to design MOFs precursors at the molecular level to synthesize Fe–N–C electrocatalysts with homogeneous distribution, high‐density active sites, and efficient mass transportation. For example, a special lamellar MOF denoted as DCI–Fe had been designed by Wu and co‐workers by a solvothermal reaction,^190^ using N‐rich dicyanoimidazole (DCI) and iron acetate as ligands and metal centers. The N‐doped carbon sheets of DCI–Fe own rich Fe and N with well dispersed active sites, and this can be attributed to the uniform distributions of N, C, and Fe at the molecular level in MOF building blocks (**Figure** **16**). The desirable multimodal pore structure is composed of mesopores resulting from the inherent lamellar morphology of MOFs and nanopores arising from the pyrolysis of MOF precursors. As a result, the Fe–N–C electrocatalysts benefiting from the above unique structures exhibited a superior electrocatalytic activity and stability compared with commercial Pt/C catalysts towards ORR in alkaline conditions.

The available MOF‐derived heteroatom‐doped carbon electrocatalysts and their corresponding ORR catalytic activities in terms of onset and half‐wave potential are summarized in **Table** **1**.

**Table 1:** The summary of the electrocatalytic activities of MOF‐derived heteroatom‐doped carbon electrocatalysts

MOF	Doped element	Electrolyte [m]	*E*_onset_ [V]	*E*_Half‐wave_ [V]	Ref.
ZIF‐8	N	0.1 KOH	−0.02 (vs. Ag/AgCl)	−0.2	87
ZIF‐7	N	0.1 KOH	0.86 (vs. RHE)	0.7	89
ZIF‐8	N	0.1 KOH	0.06 (vs. Ag/AgCl)	0.103	191
Zn_2_(TPT)(BDC)_2_	N	0.1 KOH	1.0 (vs. RHE)	0.88	192
ZIF‐8	N	0.1 KOH	0.881 (vs. RHE)	0.822	193
ZIF‐8	N	0.1 KOH	1.01 (vs. RHE)	0.8	100
MOF‐5	N	0.1 KOH	−0.051 (vs. Ag/AgCl)	0.171	194
MOF‐74	N	0.1 KOH	1.02 (vs. RHE)	0.902	195
MOF‐5	N, S	0.1 KOH	0.005 (vs. Ag/AgCl)	−0.1	114
MC‐BIF‐1S	N, B	0.1 KOH	0.894 (vs. RHE)	0.803	122
MOF‐5	N, S, P	0.1 KOH	−0.007 (vs. Ag/AgCl)	−0.06	115
ZIF‐67	Co, N	0.1 KOH	0.91 (vs. RHE)	0.85	155
ZIF‐67	Co, N	0.1 KOH	0.93 (vs. RHE)	0.83	171
ZIF‐67	Co, N	0.1 HClO_4_	0.86 (vs. RHE)	0.71	156
ZIF‐67	Co, N	0.1 KOH	0.973 (vs. RHE)	0.871	172
ZnCo‐BMOF	Co, N	0.1 KOH	0.982 (vs. RHE)	0.881	176
ZIF‐67	Co, N	0.5 H_2_SO_4_	0.845 (vs. RHE)	0.794	196
ZIF‐67	Co, N	0.1 KOH	0.92 (vs. RHE)	0.83	197
ZIF‐67	Co, N	0.1 KOH	1.016 (vs. RHE)	0.886	198
ZIF‐67	Co, N	0.1 KOH	1.033 (vs. RHE)	0.825	158
Co‐BDC	Co, N	0.1 KOH	0.90 (vs. RHE)	0.80	199
ZIF‐8@ZIF‐67	Co, N	0.1 KOH	0.93 (vs. RHE)	0.83	166
Co(mIM)_2_	Co, N, O	0.1 KOH	1.019 (vs. RHE)	0.83	200
Co–MOFs	Co, N	0.1 KOH	0.88 (vs. RHE)	0.79	201
NiCo–MOFs	Co,Ni, N	0.1 KOH	−0.051 (vs. Hg/HgO)	−0.049	150
ZIF‐8@ZIF‐67	Co, N	0.1 KOH	0.92 (vs. RHE)	0.82	128
MIL‐101‐NH_2_	Co, N, S	0.1 KOH	−0.05 (vs. Ag/AgCl)	−0.17	202
ZIF‐8@ZIF‐67	Co, N	0.1 KOH	0.926 (vs. RHE)	0.855	203
ZnCo–ZIF	Co, N	0.1 KOH	0.976 (vs. RHE)	0.849	204
ZnCo–ZIF	Co, N, O	0.1 KOH	−0.099 (vs. Ag/AgCl)	−0.2	192
ZIF‐67	Co, N	0.1 KOH	1. 017 (vs. RHE)	0.857	205
ZIF‐67	Co, N, S	0.1 KOH	0.96 (vs. RHE)	0.83	206
MIL‐101‐NH_2_	Fe, N	0.1 KOH	−0.01 (vs. Ag/AgCl)	−0.13	186
ZIF‐8	Fe, N	0.1 KOH	0.95 (vs. RHE)	0.82	187
ZIF‐8	Fe, N	0.1 KOH	0.95 (vs. RHE)	0.84	188
ZIF‐7	Fe, N	0.1 KOH	1.04 (vs. RHE)	0.88	207
DCI–Fe	Fe, N	0.1 KOH	−0.01 (vs. Ag/AgCl)	−0.18	190
MIL‐101‐Fe	Fe, Co, N	0.1 KOH	0.96 (vs. RHE)	0.82	208
HKUST‐1	Fe, N	0.1 KOH	−0.038 (vs. Ag/AgCl)	−0.175	209
Fe/Co‐MOF	Fe, Co, N	0.1 KOH	0.88 (vs. RHE)	0.79	210
ZIF‐8	Fe, N	0.1 KOH	0.96 (vs. RHE)	0.83	211
Fe/Ni‐MIL	Fe, Ni, N	0.1 KOH	1.03(vs. RHE)	0.86	212
MOF‐253	Fe, N	0.1 KOH	0.98 (vs. RHE)	0.84	213
Fe_III_‐IRMOF‐3	Fe, N	0.1 KOH	0.93 (vs. RHE)	0.78	214
MIL‐101‐Fe	Fe, N	0.1 KOH	0.04 (vs. Ag/AgCl)	−0.1	215
Fe(acac)_3_@ZIF‐8	Fe, N	0.1 KOH	1.0 (vs. RHE)	0.90	216

## 4 Summary and Outlook

Heteroatom‐doped carbon materials as efficient ORR electrocatalysts hold great promise to replace noble metal electrocatalysts for advanced electrochemical energy conversion systems including proton exchange membrane fuel cells, metal–air batteries, etc. Doping different elements, fabricating multidimensional structures and constructing of hierarchical pores have been widely employed to design superior ORR electrocatalysts. The fabrication of MOF‐derived heteroatom‐doped carbon electrocatalysts usually owns the advantages of mild reaction conditions, convenient operating process, and low cost. Furthermore, benefiting from the unique and adjustable structure of MOF precursors, the obtained electrocatalysts possess ultrahigh specific surface area, hierarchical pores structure, and high‐density active sites with a good dispersity, which will provide fast mass and proton transfers as well as the enhanced catalytic activity of ORR. The general strategy for the fabrication of nonmetal heteroatom‐doped carbon electrocatalysts from MOFs is performed through introducing guest molecules or a direct carbonation of MOF precursors containing heteroatoms. Unfortunately, they might suffer from a small amount of metal residue, thus resulting in ambiguous catalytic mechanism for metal‐free carbon‐based electrocatalysts of ORR. It has been discovered that the introduction of the additional carbon into the carbonation process of host MOFs is not only conduce to completing removal of residual metals or their compounds, but also enhances the graphitization degree of ultimate carbon‐based materials. Even so, the related research is still in the preliminary stage. Therefore, for the nonmetal heteroatom‐doped carbon electrocatalysts, the future research efforts will be advocated to solve the issue of metal residues and explore the catalytic mechanism of pure metal‐free carbon electrocatalysts derived from MOFs. In addition, the multiplex heteroatom‐doped carbon electrocatalysts could further improve electrocatalytic activity of ORR, which can be attributed to the synergistic effects among the various heteroatoms that can generate a large asymmetrical spin and optimize the charge density into carbon skeletons.

The high catalytic performance of the electrocatalysts greatly depends on their structure, and so the structure optimization is an essential study for the development of advanced electrocatalyst technologies. For MOF‐derived M–N*_x_* moieties doped carbon electrocatalysts, most of the researches have devoted to designing structures for superior performances of ORR catalysis. Molecules with special functions are usually introduced into MOF precursors to functionalize the target electrocatalysts. In addition, tuning porous size and structure to expose more active surface area, introducing heteroatom to provide more active site center, and preventing aggregation of M–N*_x_* sites to form homodispersed high‐density active sites are efficient to enhance catalytic activity of electrocatalysts for ORR. Moreover, the size dimension is also closely associated with the transport properties and electrocatalytic activities of electrocatalysts for ORR. It has been demonstrated that the electrocatalysts with smaller sizes can provide more favorable mass and electron transfer process. Nevertheless, most of synthetic routes to MOF‐derived M–N*_x_* doped carbon‐based catalysts involve high‐temperature pyrolysis, which may yield both two or multifarious metallic compound species, and this is unfavorable for the identification of exclusive role of each species in the catalysis. Therefore, this will possibly be one of the key problems to improve MOF‐derived metal‐doped carbon catalysts in the future. In addition, a grave impediment for the development of MOF‐derived carbon catalysts is prone to agglomerate and is difficult to grow on the substrate during the high‐temperature treatment, and it will possibly become another research hotspot to enhance transport kinetics and improve electrocatalytic activity of ORR. Based on the above summaries, recently numerous significant progresses of MOF‐derived heteroatom‐doped carbon electrocatalysts with high‐performance for ORR have been achieved, and it will be hopeful to create much more innovations for carbon‐based electrocatalysts in the future.

## Conflict of Interest

The authors declare no conflict of interest.
